# Chronic Conditions, Persistent Pain, and Psychological Distress Among the Rural Older Adults: A Path Analysis in Shandong, China

**DOI:** 10.3389/fmed.2021.770914

**Published:** 2021-11-02

**Authors:** Shijun Yang, Jie Li, Dan Zhao, Yi Wang, Wenjuan Li, Jie Li, Zhixian Li, Zhen Wei, Chen Yan, Zhen Gui, Chengchao Zhou

**Affiliations:** ^1^Centre for Health Management and Policy Research, School of Public Health, Cheeloo College of Medicine, Shandong University, Jinan, China; ^2^National Health Committee (NHC) Key Lab of Health Economics and Policy Research, Shandong University, Jinan, China

**Keywords:** psychological distress, chronic conditions, persistent pain, older adults, rural

## Abstract

Psychological distress were found to be associated with chronic conditions and persistent pain. However, few studies explored the underlying pathways between them. This study aimed to analyze the path of chronic conditions and persistent pain on psychological distress through sleep quality and self-rated health. A total of 2,748 rural older people in Shandong, China were included in this study. Path analysis was performed by using Mplus 8.3 to examine the associations between chronic conditions, persistent pain, sleep quality, self-rated health, and psychological distress after adjusting for age, gender, education, and household income. The prevalence of psychological distress among the older adults in this study was 47.49%. Chronic conditions and persistent pain were indirectly associated with psychological distress through six mediating pathways: (1) the path from chronic conditions to psychological distress through sleep quality (β = 0.041, 95%CI: 0.015–0.067) and self-rated health (β = 0.064, 95%CI: 0.038–0.091), respectively, and a chain mediation existed (β = 0.007, 95% CI: 0.000–0.014); (2) the path of persistent pain and psychological distress through sleep quality (β = 0.058, 95% CI: 0.014–0.102) and self-rated health (β = 0.048, 95% CI: 0.000–0.096), respectively, also the chain mediation found (β = 0.009, 95% CI: 0.005–0.014). Psychological distress was associated with chronic conditions and persistent pain through decreased sleep quality and self-rated health among Chinese rural older people. Multi-pronged targeted intervention should be taken for older adults with chronic conditions and persistent pain.

## Introduction

Population aging is accelerating worldwide, especially in China, where the total older population will exceed 400 million and the proportion of older adults will exceed 30% by 2050 (1). Meanwhile, there were more than one-third of the Chinese older population had a high risk of severe psychological distress (PD) symptoms (2). PD is defined as a state of emotional distress, characterized by depression, and anxiety symptoms. It is widely employed to reflect the mental health of people (3). Previous studies found that rural older adults had worse mental health than those in urban areas (4, 5). There were 38.8% of Chinese rural seniors had a high degree of PD, which was higher than the rate of urban older adults (22.2%) (2). Previous studies demonstrated that PD was associated with a high risk of suicide ideas, plans, and behaviors, especially among the older population (6–8). PD was also related to all-cause mortality (9). Exploring risk factors and their pathways to PD is critical to improving mental health, preventing adverse health outcomes, and reducing mortality.

Among the risk factors of PD, chronic conditions, and persistent pain were common among the older population (2, 10–12). The status that one person with two or more chronic conditions was defined as multimorbidity (13, 14), which was found to be a risk factor of PD among older adults. According to previous studies, multimorbid older adults had higher PD levels compared to those without multimorbidity (11, 15). Persistent pain is another important risk factor for mental health among older adults (16). Previous studies found that there was a higher risk of PD for older people with persistent pain (10, 17, 18). Additionally, chronic conditions and persistent pain often coexisted among numerous older people. Persistent pain was frequently interwoven with other physical or mental health problems (19, 20). The relationship between chronic conditions and PD, persistent pain and PD, have been demonstrated, respectively, in previous studies. But the potential associations are still unclear, and the association with PD when chronic conditions and persistent pain coexist has not been explored.

Psychological distress theory has been proposed by many scholars (21–23). It could be concluded that stressors (e.g., physical disorders or life events) stimulate the body through mediating mechanisms (e.g., stress perceiving and cognitive evaluation), and result in mental health problems [e.g., psychological distress; (22)]. According to this theory, chronic stress such as chronic conditions and persistent pain could be regarded as stressors (24). They were also found to be related to decreased sleep quality and worse self-rated health (25–28). It is considerable that sleep quality and self-rated health might play mediating roles in the associations between stressors and PD (22). Poor self-rated health was found to be associated with higher odds of PD among older adults (25, 29). Several studies had revealed that the number of chronic conditions had a strong association with self-rated health among older people (26, 30). Furthermore, a study conducted among older Chinese and older Korean Americans indicated that self-rated health played a mediating role between chronic conditions and depressive symptoms in both groups (31). Thus, self-rated health might be a mediator in the relationship between chronic conditions and PD in this study. Also, persistent pain was another risk factor related to poor self-rated health according to previous studies (26, 27, 32–34). As such, self-rated health might be one mediator for persistent pain and PD.

Sleep quality could be another mediator on the basis of psychological distress theory (22). There were significant correlations between mental disorders and sleep problems among older adults (28, 35, 36). As for chronic conditions, high prevalence of chronic diseases has been found to be associated with poor sleep quality (37, 38). Therefore, sleep quality may play a mediating role in the relationship between chronic conditions and PD. Further, previous research has indicated that persistent pain may result in sleep disturbance among older people (27, 39). It has been found that pain intensity was associated with sleep-related issues (40). The relationship between pain catastrophizing and depression among older Korean adults had been explored, and sleep quality was one of the mediators (41). Consequently, sleep quality could be one mediator for the relationship between persistent pain and PD.

**Figure 1:**
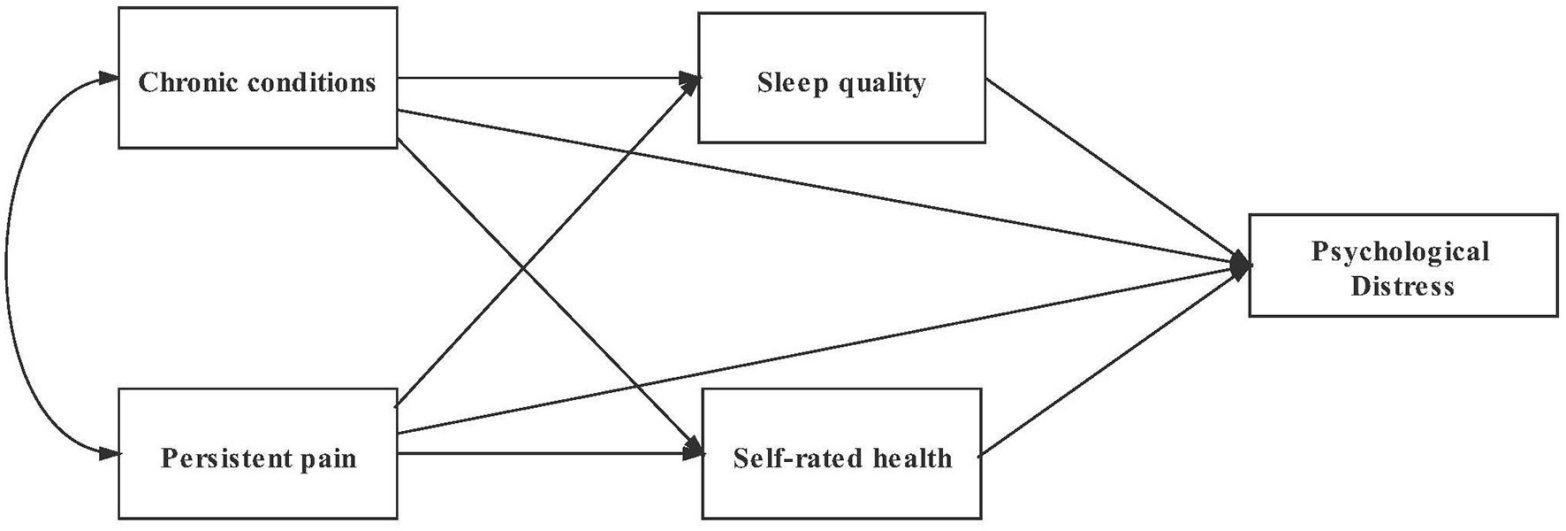
Hypothesized model.

Based on the psychological distress theoretical framework and previous evidence, we make the following hypotheses: chronic conditions and persistent pain were regarded as the stressors; sleep quality and self-rated health were treated as mediating factors, and the psychological response was the appearance of PD. The hypothesized model of this study included six pathways (Figure 1). The mediating effects of sleep quality and self-rated health would be examined. In addition, some researches had shown that sleep quality was associated with self-rated health among people over 18 years old (42, 43). We would include the relationship in our analysis, to confirm whether the chain mediating effects exist.

## Materials and Methods

### Study Design and Sample

This study was based on the Shandong Rural Elderly Health Cohort (SREHC), an ongoing longitudinal study conducted in the second-most populous province in China. SREHC takes the population of 60 years old and above in rural Shandong as the research subjects, aims to investigate seniors' health status, and deal with the problem of population aging. The baseline survey was conducted from May to June in 2019, and the follow-up survey was conducted from August to September in 2020. In this study, we used the follow-up data.

**Figure 2:**
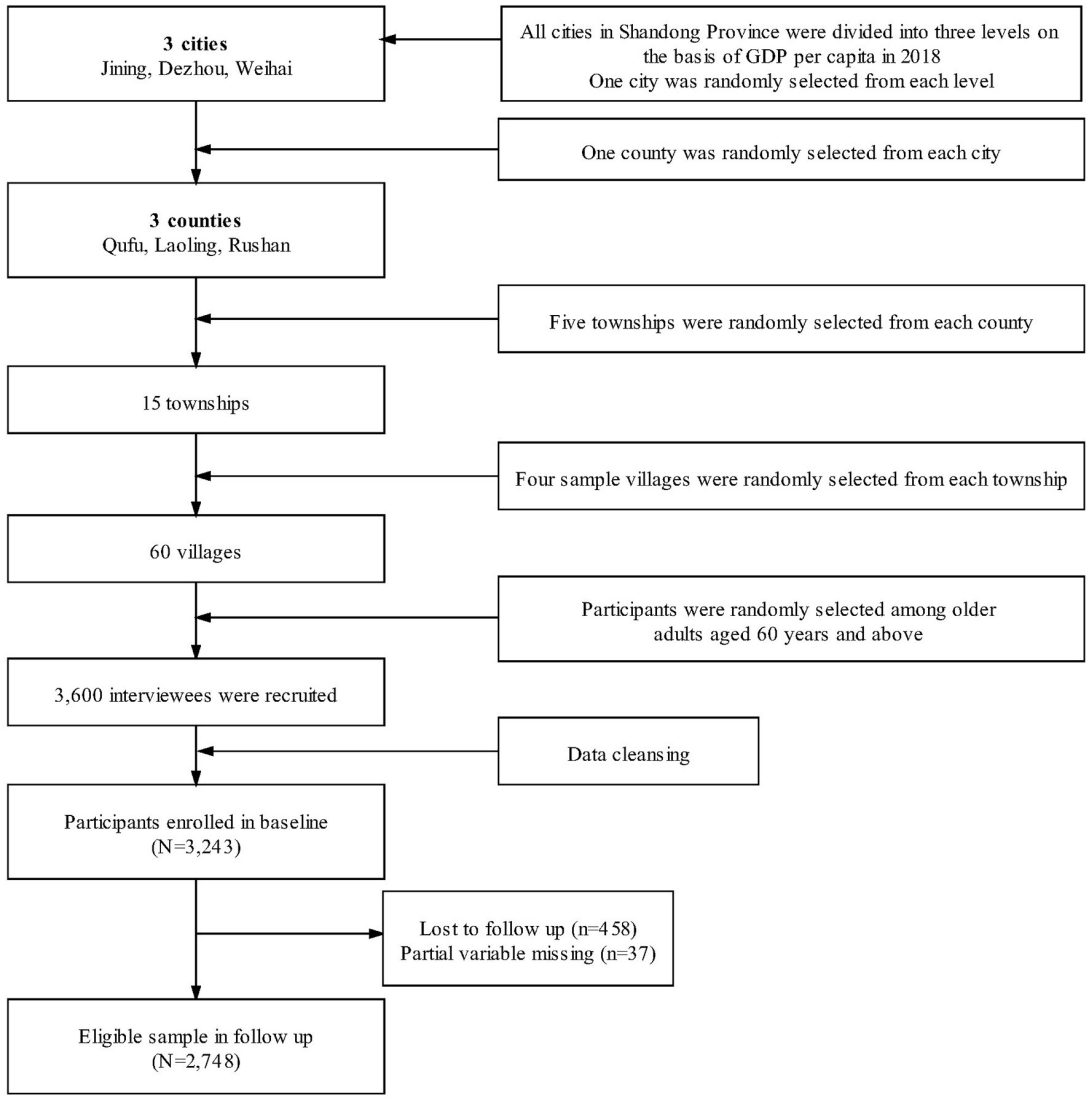
Flowchart of participants selection in this study.

A three-stage stratified random sampling method was employed to select participants at baseline (Figure 2). Firstly, all counties of Shandong province were divided into three groups on the basis of GDP per capita (2018). Secondly, one county was selected randomly from each group. Three counties (Rushan, Qufu, Laoling, respectively, represented high, medium, and low level county) were chosen as the study sites. After that, five townships were randomly selected from each sampled county. Thirdly, four villages were chosen randomly from each township. Older adults aged 60 years and above were investigated in each sample village. In total, 3,600 individuals were recruited and 3,243 completed the whole survey, with a response rate of 90.05% (44). Among the 3,243 respondents in the baseline survey, 2,785 participated in the follow-up survey in 2020 (45). After excluding 37 respondents whose core variables were missing, we finally included 2,748 respondents in this study. Of all basic characteristics and core variables, there was no significant difference between the respondents included in this study and those not.

We collected information through a structured questionnaire. A face-to-face survey was conducted by well-trained investigators in both two surveys. Completed questionnaires were carefully checked by the supervisors after the interview each day to ensure quality. Ethical approval was received from the Institutional Review Board at the authors' University.

### Measures

#### Chronic Conditions

In this study, we used the number of chronic diseases to represent chronic conditions. It was categorized into three groups: no chronic condition, one chronic condition, and multimorbidity (chronic conditions ≥2) (12). According to the classification of World Health Organization (46), 12 types of chronic disease were considered, including hypertension, dyslipidemia, diabetes or elevated blood sugar, malignant tumors, chronic lung disease, liver disease, heart disease, stroke, kidney disease, stomach or digestive system disease, arthritis or rheumatism, and asthma.

#### Persistent Pain

The persistent pain was measured by the question “Do you feel pain for a long time (longer than 6 months or more)?” The answer included “yes” and “no” (47). This tool was widely used to evaluate the prevalence of persistent pain (48).

#### Self-Rated Health

Self-rated health was measured by a question of “In general terms, how would you assess your health status?” The answer included “good,” “quite good,” “normal,” “rather poor,” and “poor.” The respondents with good or quite good self-rated health were classified as having good self-rated health, and those with rather poor or poor were classified as having poor self-rated health (32). It was a widely used predictor to assess subjective health (49).

#### Sleep Quality

We used the Chinese version of the Pittsburgh Sleep Quality Index (PSQI), which has good reliability and validity (50). This scale consists of 19 items and is categorized into 7 dimensions, including sleep duration, sleep disturbance, sleep latency, daytime dysfunction, sleep efficiency, overall sleep quality, and use of sleeping medication. Each of these is scored from 0 to 3. The total PSQI score ranged from 0 to 21, higher scores indicated worse sleep quality (51). The respondents' PSQI score <7 represented good sleep quality; those ≥7 indicated poor sleep quality (52).

#### Psychological Distress

Psychological distress was examined by Kessler Psychological Distress Scale (K10), which has been widely used to assess psychological health for older adults (3), and the Chinese version of K10 has been confirmed to have good reliability and validity (53). The scale mainly focuses on the PD of the respondents in the past 30 days. It contained 10-items and each item contained a 5-point Likert item. The total score for the scale ranged from 10 to 50 points, higher scores represented a higher risk of mental disorder. Finally, it was divided into 3 levels: no/low level, mild level, and moderate/severe level (53, 54).

#### Covariates

Sociodemographic characteristics included age, gender, marital status, education, and economic status. In this study, age was divided into 60–, 70–, and 80+ years, gender included male and female, education included illiteracy or semiliterate, primary school, junior school, and senior school or above. Economic status was estimated by household income of last year. Quartile 1 was the poorest and Quartile 4 was the richest.

### Statistical Analysis

SPSS 24.0 (IBM, New York, NY, USA) and Mplus 8.3 (Muthén & Muthén, Los Angeles, CA, USA) were used to analyze the data. Firstly, frequency and percentage were used to describe the demographic characteristics of the respondents. Then we used the Chi-square tests and Kruskal-Wallis tests to compare the differences of PD degrees across different subgroups. Secondly, Spearman's correlation analysis and Chi-square tests were used to examine the correlation among all variables. Finally, we used Mplus 8.3 to test the hypothesized models with and without the pathway of sleep quality to self-rated health, respectively. Based on the results of univariate analysis and previous studies, the basic characteristics related to PD and mediators were, respectively, regarded as their covariates. Past research typically had shown that demographic characteristics (including age, gender, marital status, education, and economic status) were associated with sleep quality, self-rated health, and PD, respectively (14, 32, 37, 41). The WLSMV estimator was employed in this model because it has been found to perform well for path analysis with ordinal or categorical dependent variables (55, 56). The fit index and its acceptable value of the model including Normed Chi-square (χ^2^/*DF* <5), root mean square error of approximation (RMSEA <0.08), standardized root mean square residual (SRMR <0.05), Tucker-Lewis index (TLI), and comparative fit index (CFI >0.90) (57, 58). All statistical analyses set a statistically significant threshold with *P* < 0.05.

## Results

### Characteristics of Participants

The percentages of respondents identified as no/low, mild, and moderate/severe PD were 52.51% (*n* = 1,443), 27.77% (*n* = 763), and 19.72% (*n* = 542), respectively (Table 1). There were 1,305 (47.49%) respondents who had mild and above degrees of PD. Of the 2,748 respondents, 1,745 were female (63.50%), 2,026 have been married (73.73%), and 1,143 were illiterate (41.59%). The majority of them had more than one chronic condition (75.15%), without persistent pain (58.33%), had poor sleep quality (67.21%), and had good self-rated health status (58.33%).

**Table 1:** Characters associated with psychological distress among the older adults in Shandong, China, 2020.

**Variable**	***N* (%)/($\bar{x}\pm s$)**	**Psychological distress**				
		**No/Low**	**Mild**	**Moderate/Severe**	***χ*^2^/H**	***P*-Value**
**Observations**	2,748	1,443 (52.51)	763 (27.77)	542 (19.72)		
**Age**					2.96	0.57
60–	1,175 (42.76)	624 (43.24)	326 (42.73)	225 (41.51)		
70–	1,301 (47.34)	670 (46.43)	372 (48.75)	259 (47.79)		
80+	272 (9.90)	149 (10.33)	65 (8.52)	58 (10.70)		
**Gender**					58.45	<0.001
Male	1,003 (36.50)	618 (42.83)	246 (32.24)	139 (25.65)		
Female	1,745 (63.50)	825 (57.17)	517 (67.76)	403 (74.35)		
**Education**					24.16	<0.001
Illiteracy or semiliterate	1,143 (41.59)	550 (38.12)	326 (42.73)	267 (49.26)		
Primary school	1,065 (38.76)	556 (39.22)	298 (39.06)	201 (37.08)		
Junior school	403 (14.67)	240 (16.63)	107 (14.02)	56 (10.33)		
Senior school or above	137 (4.99)	87 (6.03)	32 (4.19)	18 (3.32)		
**Household income**					39.29	<0.001
Q1 (Poorest)	673 (24.49)	289 (20.03)	214 (28.05)	170 (31.37)		
Q2	700 (25.47)	378 (26.20)	195 (25.56)	127 (23.43)		
Q3	686 (24.96)	352 (24.39)	199 (26.08)	135 (24.91)		
Q4 (Richest)	689 (25.07)	424 (29.38)	155 (20.31)	110 (20.30)		
**Self-rated health**					164.07	<0.001
Good	1,603 (58.33)	1,012 (70.13)	359 (47.05)	232 (42.80)		
Normal	689 (25.07)	302 (20.93)	227 (29.75)	160 (29.52)		
Poor	456 (16.59)	129 (8.94)	177 (23.20)	150 (27.68)		
**Chronic conditions**					52.16	<0.001
No chronic condition	683 (24.85)	443 (30.70)	139 (18.22)	101 (18.63)		
One chronic condition	945 (34.39)	501 (34.72)	261 (34.21)	183 (33.76)		
Multimorbidity	1,120 (40.76)	499 (34.58)	363 (47.58)	258 (47.60)		
**Persistent pain**					88.97	<0.001
Yes	1,145 (41.67)	482 (33.40)	371 (48.62)	292 (53.87)		
No	1,603 (58.33)	961 (66.60)	392 (51.38)	250 (46.13)		
**Sleep quality**					172.06	<0.001
PSQI <7	901 (32.79)	628 (43.52)	190 (24.90)	83 (15.31)		
PSQI ≥7	1,847 (67.21)	815 (56.48)	573 (75.10)	459 (84.69)		

### Bi-Variate Correlation of Variables

The correlation matrix of the main variables and covariates is shown in Table 2. Persistent pain (χ^2^ = 88.973, *P* < 0.001), chronic conditions (*r* = 0.143, *P* < 0.001), self-rated health (*r* = 0.260, *P* < 0.001), and sleep quality (χ^2^ = 117.333, *P* < 0.001) were all positively related to PD. Older adults with persistent pain, more than one chronic condition, poor self-rated health status, and poor sleep quality were more likely to have severer PD. Age, gender, education, and household income were considered as covariates. The association between covariates and sleep quality, self-rated health, and PD were also shown in Table 2.

**Table 2:** Bi-variate correlation of variables (*N* = 2,748).

**Variables**	**1^**a**^**	**2**	**3**	**4^**a**^**	**5**
1. Persistent pain^a^	1.000				
2. Chronic conditions	191.666^***^	1.000			
3. Self-rated health	261.585^***^	0.309^***^	1.000		
4. Sleep quality^a^	117.333^***^	86.656^***^	104.775^***^	1.000	
5. Psychological distress	88.973^***^	0.143^***^	0.260^***^	172.057^***^	1.000
6. Age	2.735	−0.021	0.006	6.291^*^	0.009
7. Gender^a^	25.572^***^	14.974^**^	19.174^***^	102.837^***^	58.448^***^
8. Education	8.488^*^	−0.023	−0.043^*^	39.847^***^	−0.104^***^
9. Household income	6.065	−0.032	−0.063^***^	16.535^**^	−0.112^***^

a *Persistent pain, sleep quality, and gender are categorical variables, χ^2^-test was employed to analyze the correlation between them and other variables*.

*** *P < 0.001*.

** *P < 0.01*.

* *P < 0.05*.

### Path Analysis

**Figure 3:**
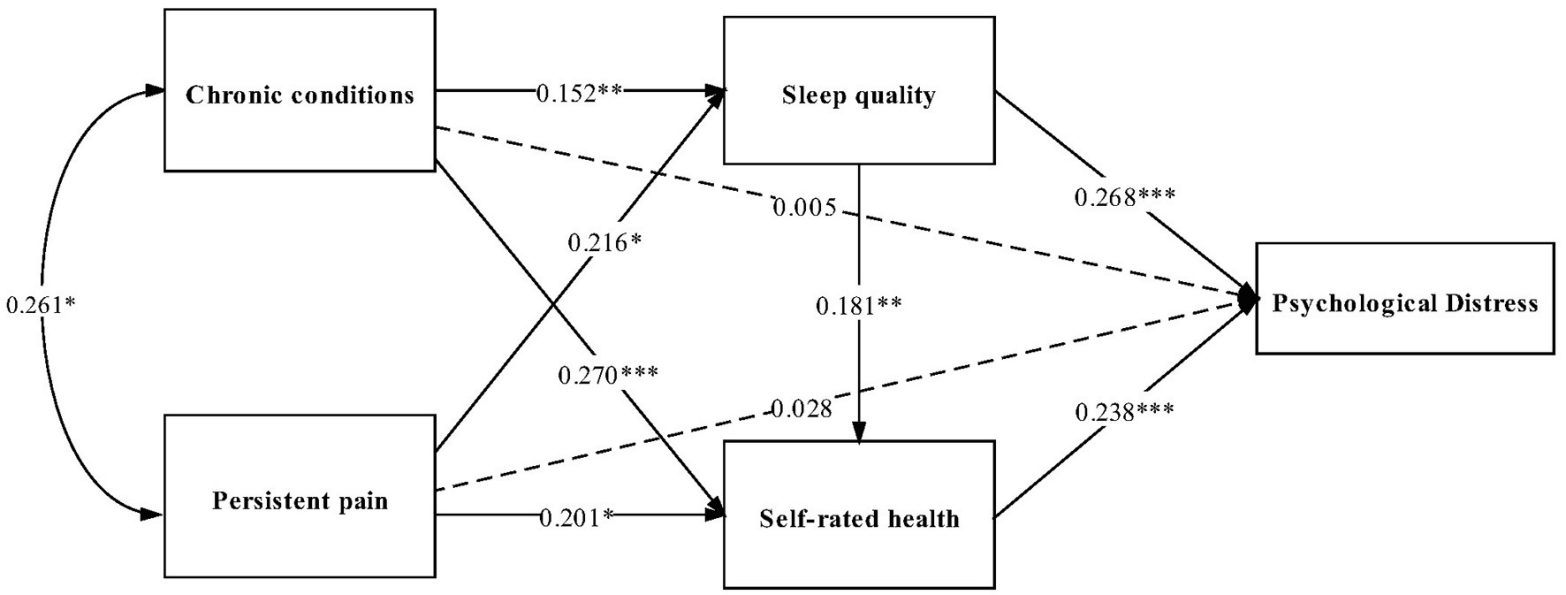
Path analysis of psychological distress among the older adults in Shandong, China, 2020. Standard path coefficients are shown. ***P < 0.001, **P < 0.01, and *P < 0.05.

The hypothesized path analysis model was tested (Figure 3). It had the Normed Chi-square of 1.95, the RMSEA was 0.019, the TLI was 0.988, the CFI was 0.957, and the SRMR was 0.045, which presented a good global fit.

As shown in Table 3, no significantly direct effect was found between chronic conditions and PD, while the significantly indirect effect of sleep quality (Estimate = 0.041, 95%CI: 0.015–0.067) and self-rated health (Estimate = 0.064, 95%CI: 0.038–0.091), respectively, existed in the association of chronic conditions and PD. The chain mediation of sleep quality and self-rated health on chronic conditions and PD were significant (Estimate = 0.007, 95%CI: 0.000–0.014). The total mediating effect of chronic conditions on PD was 95.73% (Estimate = 0.112, 95%CI: 0.058–0.166). For persistent pain, it had no significant direct effect on PD. Sleep quality (Estimate = 0.058, 95%CI: 0.014–0.102) and self-rated health (Estimate = 0.048, 95%CI: 0.000–0.096), respectively, played the mediating roles between persistent pain and PD. Additionally, sleep quality and self-rated health acted as chain mediators between persistent pain and PD (Estimate = 0.009, 95%CI: 0.005–0.014). The total indirect effect of persistent pain through sleep quality and self-rated health on PD was 80.42% (Estimate = 0.115, 95%CI: 0.023–0.207).

**Table 3:** Standardized effects on psychological distress from path analysis among the older adults in Shandong, China, 2020.

**Model pathways**	**Estimate**	**S.E**.	**95% C.I**.
Total effect chronic conditions → Psychological distress	0.117^**^	0.042	0.048–0.186
Direct effect chronic conditions → Psychological distress	0.005	0.023	−0.033 to 0.044
Total indirect effect chronic conditions → Psychological distress	0.112^**^	0.033	0.058–0.166
Chronic conditions → Sleep quality → Psychological distress	0.041^**^	0.016	0.015–0.067
Chronic conditions → Self-rated health → Psychological distress	0.064^***^	0.016	0.038–0.091
Chronic conditions → Sleep quality → Self-rated health → Psychological distress	0.007^*^	0.004	0.000–0.014
Total effect persistent pain → Psychological distress	0.143^*^	0.084	0.006–0.281
Direct effect persistent pain → Psychological distress	0.028	0.035	−0.029 to 0.085
Total indirect effect persistent pain → Psychological distress	0.115^*^	0.056	0.023–0.207
Persistent pain → Sleep quality → Psychological distress	0.058^*^	0.027	0.014–0.102
Persistent pain → Self-rated health → Psychological distress	0.048^*^	0.029	0.000–0.096
Persistent pain → Sleep quality → Self-rated health → Psychological distress	0.009^***^	0.003	0.005–0.014

*** *P < 0.001*.

** *P < 0.01*.

* *P < 0.05*.

## Discussion

By using path analysis, chronic conditions and persistent pain, respectively, had indirect positive associations with PD through sleep quality and self-rated health. Meanwhile, the chain mediation of sleep quality and self-rated health existed. The hypothesis model was partially verified. It is crucial for older people to keep in good mental health, while the influencing factors of mental health are diverse and the mechanism is complex. This study explores the pathways of risk factors on PD, and provides systematic inspiration for mental health management and intervention among older adults. The highlight is that not only the mediation of sleep quality and self-rated health exist, but also the chain mediations are found.

### Chronic Conditions to PD

Older adults who suffered one chronic condition or multimorbidity had higher odds of PD than those without chronic conditions. There were three pathways between chronic conditions and PD. Older adults with chronic conditions were more likely to have PD through poor sleep quality. In accordance with other studies, people with multimorbidity reported more PD symptoms, and trouble sleeping had the greatest effect size among all depressive items (11). A cross-sectional study showed a dose-dependent correlation between the number of chronic diseases and the sleep disturbances index (59). Sleep disorders had been demonstrated to precipitate mental health problems among older adults (28). Further, older adults with chronic conditions were more likely to have PD through poor self-rated health. Previous studies showed that the number of chronic conditions was one of the determinants of self-rated health among the older population (30). The result was supported by a study finding that the relationship between physical impairments and depression in the elderly was mediated by self-rated health (60). Older adults with chronic conditions had a worse subjective perception of physical health, which in turn affects their mental health status (15, 60). Additionally, older people with chronic conditions were more likely to have PD through poor sleep quality and poor self-rated health. Sleep disorders were common in many chronic conditions, as well as occurred in populations with mental health problems (61). Preliminary evidence indicated that poor sleep quality was associated with poor self-rated health (62, 63). A study conducted among U.S. Hispanic older adults reported that sleep quality was associated with chronic conditions, self-rated physical health and mental health, and self-rated happiness (64). The specific path of those variables was first explored in our research. Older people with chronic conditions would have poor sleep quality, which might exacerbate negative thoughts, then influenced their mental health status, even increased the risk of suicide (65). The potential biological mechanism indicated that some chronic diseases may cause changes in the brain regions and neurotransmitters that control sleep, as well as the drugs for controlling the chronic symptoms influenced the sleep quality (61). Once sleep disorders appeared, the self-rated health status might be affected, and a series of PD symptoms such as confusion, frustration, or depression would aggravate (61, 64). The total mediating effect of chronic conditions on PD was 95.73%, which indicated that our mediators played critical roles to explain the relationship between chronic conditions and PD.

### Persistent Pain to PD

Results from our study indicated that older adults who suffered persistent pain had higher odds of PD than those without lasting pain. There were three pathways between persistent pain and PD. Older adults with persistent pain were more likely to suffer PD through poor sleep quality. Consistent with previous studies, insomnia, and poor sleep quality were highly correlated with persistent pain especially in female subjects (66, 67). A person's sleep amount strongly affected the symptoms of mental disorders. Sleep disorders like deprivation and disruption can result in psychotic states of paranoia, hallucinations, or mania among vulnerable groups (61). Moreover, older adults with persistent pain were more likely to have PD through poor self-rated health. A Chinese study consistent with our finding that self-rated health and perceived social support in series partly explained the association between persistent pain and depression (68). According to the cognitive-behavioral mediation model, persistent pain might bring about a negative bias of personal health perceptions, which could lead to emotional distress such as depression (18). Furthermore, our findings revealed that older people with persistent pain were more likely to have PD through poor sleep quality and poor self-rated health. Pain was related to the feelings of losing independence and autonomy. People would be influenced by the realization of physical or mental limitations when their routine life changed, which may lead to poorer self-rated health (69). While poor self-rated health was associated with PD (68, 69). Besides, the biological mechanism of pain and mental disorders had already been demonstrated. Chronic pain stimulation resulted in a series of changes within the brain, which will produce anxiety and depression motivations or pain-related behaviors, and then mental illness appeared (70). There was a study revealed that Neuroticism and maladaptive coping strategies were the mediation between the serotonin transporter gene-linked polymorphic region (5HTT-LPR) and symptoms of anxiety and depression among elite athletes (71). This genetic polymorphism could be similar among aging population. The total indirect effect of persistent pain on PD was 80.43%, which presented that sleep quality and self-rated health were non-negligible in explaining the potential association between chronic conditions and PD.

To our knowledge, this is the first study to comprehensively investigate the underlying association of chronic conditions and PD, persistent pain and PD, as well as the mediating effect of self-rated health and sleep quality on this relationship among the Chinese rural older people. It provides a scientific basis for early detection and prevention of PD, especially for the key group of health care management, which is of high theoretical and practical significance. Thus, it is necessary to strengthen the prevention of PD among rural elderly. First of all, older adults with chronic conditions, especially with multimorbidity and persistent pain, should be regarded as the key group for regular mental health surveillance and counseling services, mental health education, and other various effective intervention measures should be carried out. Furthermore, considering the mediating effects of sleep quality and self-rated health, more attention should be paid to the regular screening of sleep quality and health status evaluation, so as to resolve the older adults' sleep problems and improve their self-rated health status.

This study had several limitations. Firstly, the causal relationships between variables were still unclear. Because this is a cross-sectional study, we did not include “persistent pain” in our baseline survey so that we used the follow-up data. Prospective studies were needed to verify the effects of chronic conditions, persistent pain, sleep quality, self-rated health on PD in further investigation. Secondly, recall bias might exist because the main variables we used were self-reported. Thirdly, we only investigated the relationship between the number of chronic diseases and PD, and did not consider the duration and severity of chronic conditions. Previous study showed that the duration and severity of chronic conditions were associated with PD (72). And we did not include the pain intensity and functional limitations in this study, which were also related to PD symptoms (18, 39). These variables will be considered in further study.

Nearly half of the rural elderly reported at least mild PD in this study. Among rural older people, sleep quality, and self-rated health were found to play partial mediating roles in the association of both chronic conditions and PD, persistent pain and PD. Efforts should be taken to control the prevalence of PD. When intervening for older adults with chronic conditions and persistent pain, sleep, and self-rated health could be considered as the key targets of intervention.

## Data Availability Statement

The raw data supporting the conclusions of this article will be made available by the authors, without undue reservation.

## Ethics Statement

The studies involving human participants were reviewed and approved by Institutional Review Board at Shandong University. The patients/participants provided their written informed consent to participate in this study. Written informed consent was obtained from the individual(s) for the publication of any potentially identifiable images or data included in this article.

## Author Contributions

CZ participated in conceptualization, writing—review and editing, funding acquisition, and supervision. JL (2nd author), YW, and ZW were responsible for methodology. DZ, WL, JL (6th author), ZL, CY, and ZG participated in formal analysis and investigation. SY participated in investigation and writing—original draft preparation. All authors have read and approved the final manuscript.

## Funding

This work was supported by the National Natural Science Foundation of China (71774104, 71473152, and 71974117), the China Medical Board (16-257), Cheeloo Youth Scholar Grant, Shandong University (IFYT1810 and 2012DX006), and NHC Key Laboratory of Health Economics and Policy Research (NHC-HEPR2019014).

## Conflict of Interest

The authors declare that the research was conducted in the absence of any commercial or financial relationships that could be construed as a potential conflict of interest.

## Publisher's Note

All claims expressed in this article are solely those of the authors and do not necessarily represent those of their affiliated organizations, or those of the publisher, the editors and the reviewers. Any product that may be evaluated in this article, or claim that may be made by its manufacturer, is not guaranteed or endorsed by the publisher.
